# Supplementary figures and images for: XRN1 Is a Species-Specific Virus Restriction Factor in Yeasts
Source: PLoS Pathog. 2016 Oct 6;12(10):e1005890. doi: 10.1371/journal.ppat.1005890 (PMC5053509; doi:10.1371/journal.ppat.1005890)

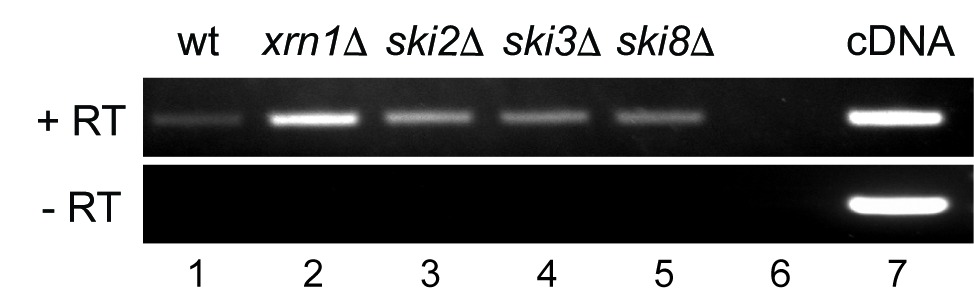

Supplement: S1 Fig — We wished to confirm that the dsRNA being detected in Fig 2A of the paper was actually L-A in origin. dsRNA samples were used as templates for L-A negative-strand-specific cDNA synthesis and subsequent PCR amplification. Specificity of our primers for L-A was confirmed by a positive control where plasmid cloned L-A cDNA was used as a template (lane 7) and a negative control where RNA extracted from S. cerevisiae 2405 (L-A-, M-) was used as a template (lane 6). (TIF) [file ppat.1005890.s001.tif]

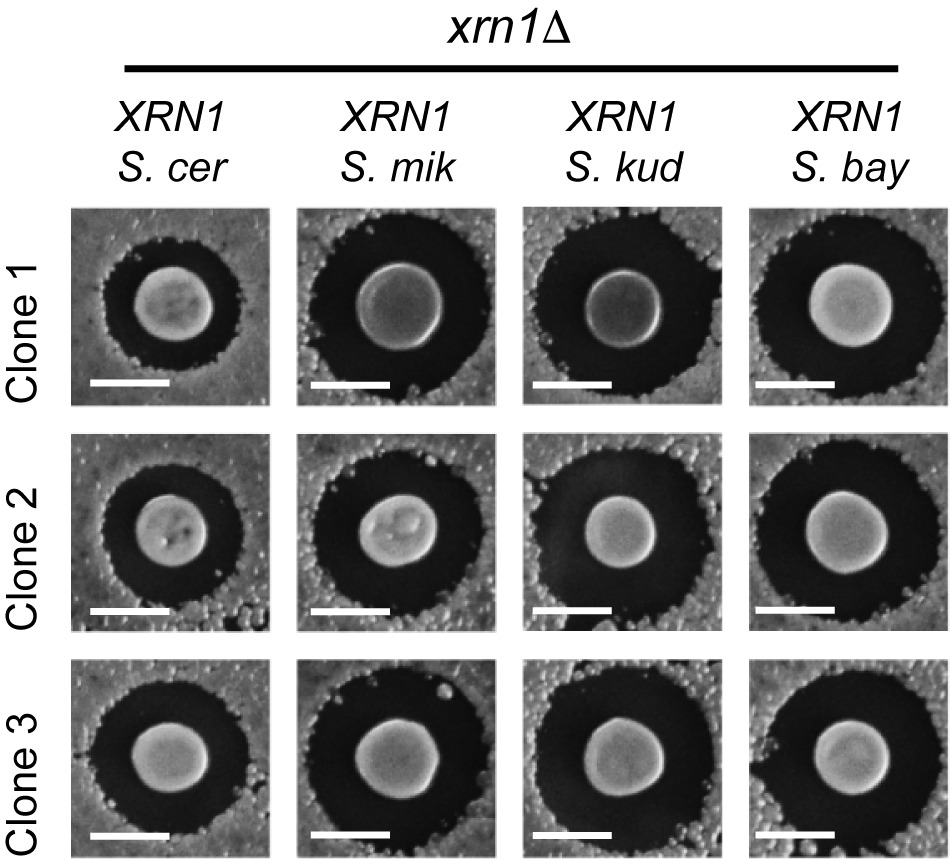

Supplement: S2 Fig — Representative pictures of kill zones produced by individual clones of S. cerevisiae xrn1Δ L-A+ Killer+ expressing XRN1 from different species. The scale bar represents 5 mm. (TIF) [file ppat.1005890.s002.tif]

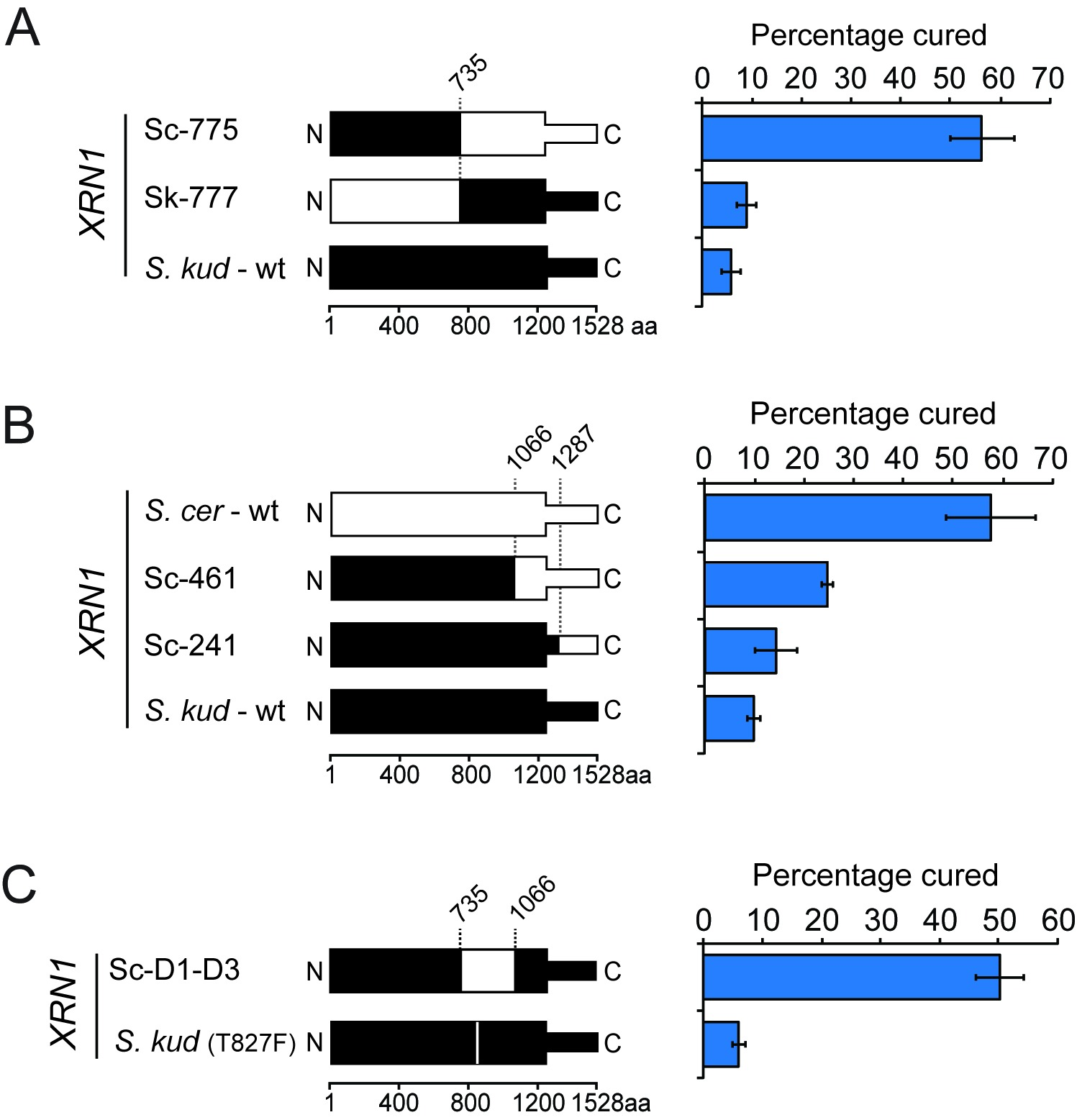

Supplement: S3 Fig — (Left) Schematic representations of chimeric proteins derived from various C-terminal domain fusions between XRN1 from S. cerevisiae (white) and S. kudriavzevii (black). Dotted lines represent the boundaries of the chimeric fusions with the numbering representing the amino acid position. (Right) Clonal isolates of a killer S. cerevisiae strain expressing chimeric Xrn1p proteins were assayed for loss of the killer phenotype resulting in “cured” clones (error bars represent SEM, n>3). (TIF) [file ppat.1005890.s003.tif]

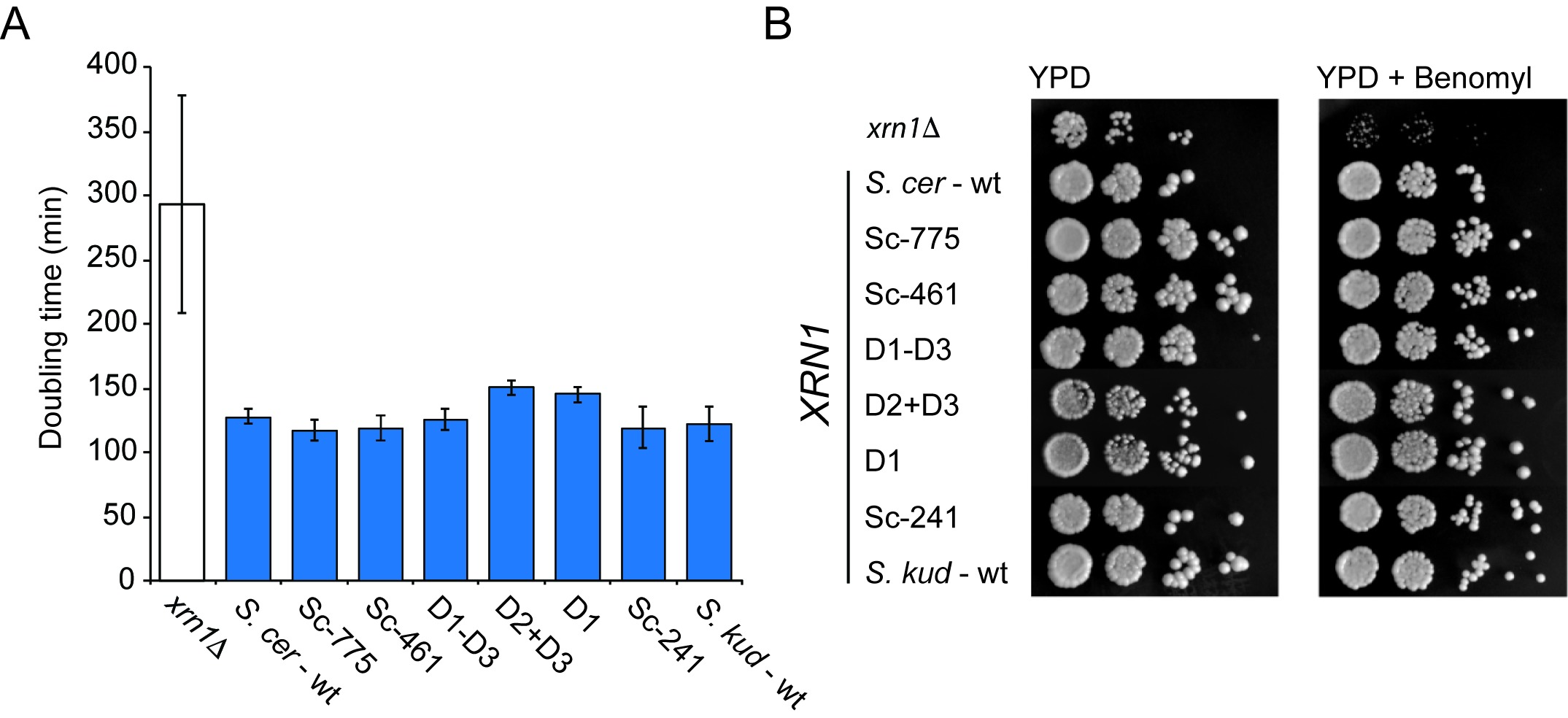

Supplement: S4 Fig — (A) The doubling time of S. cerevisiae xrn1Δ complemented with Saccharomyces XRN1 chimeras and XRN1 from S. cerevisiae and S. kudriavzevii. (B) The growth, morphology, and benomyl sensitivity of S. cerevisiae xrn1Δ cultured on YPD solid media, and the effect of complementation with XRN1 chimeras. (TIF) [file ppat.1005890.s004.tif]

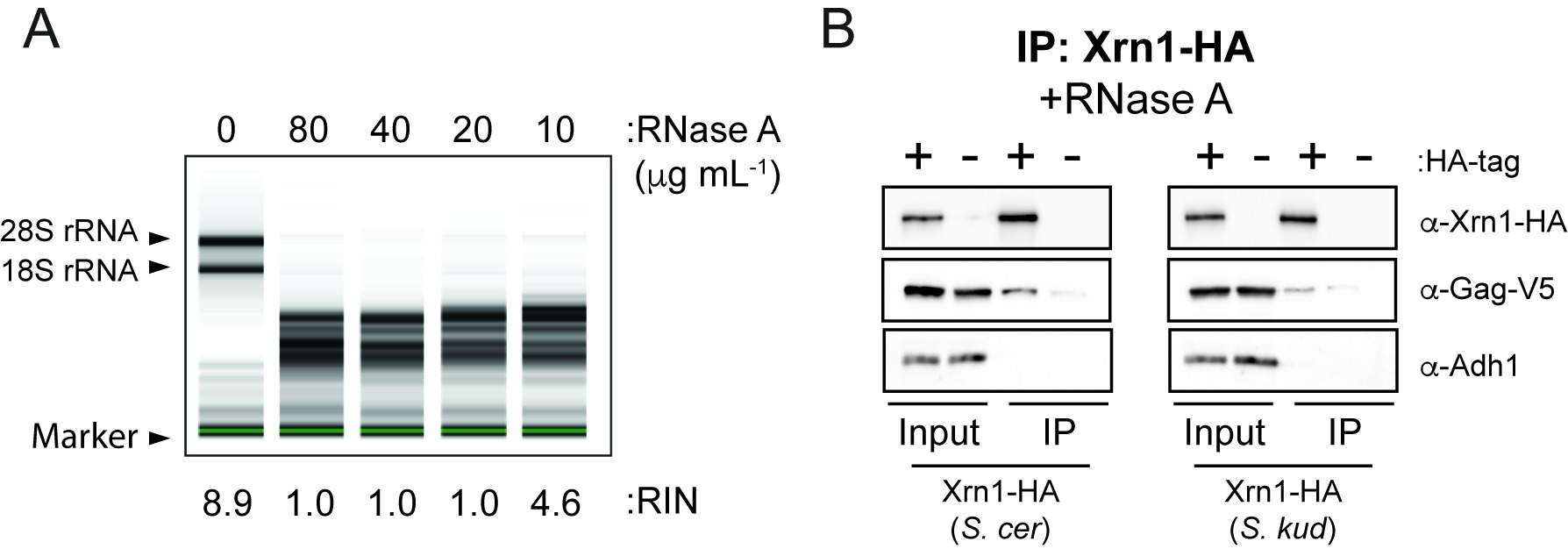

Supplement: S5 Fig — (A) The extent of RNA degradation by RNase A in yeast whole cell lysates was measured using a 2200 TapeStation Instrument with different concentrations of RNase A. RNA integrity numbers (RIN) were calculated to assess the integrity of the RNA within whole protein extract samples with and without the addition of RNase A [93]. (B) Western blot analysis of Xrn1p and L-A Gag co-immunoprecipitation. HA-tagged and untagged Xrn1p from either S. cerevisiae or S. kudriavzevii were immunoprecipitated in the presence of Gag-V5 with the addition of RNase A. Adh1 was used in all panels as a loading control to ensure equal input of total protein and the specificity of immunoprecipitation. (TIF) [file ppat.1005890.s005.tif]

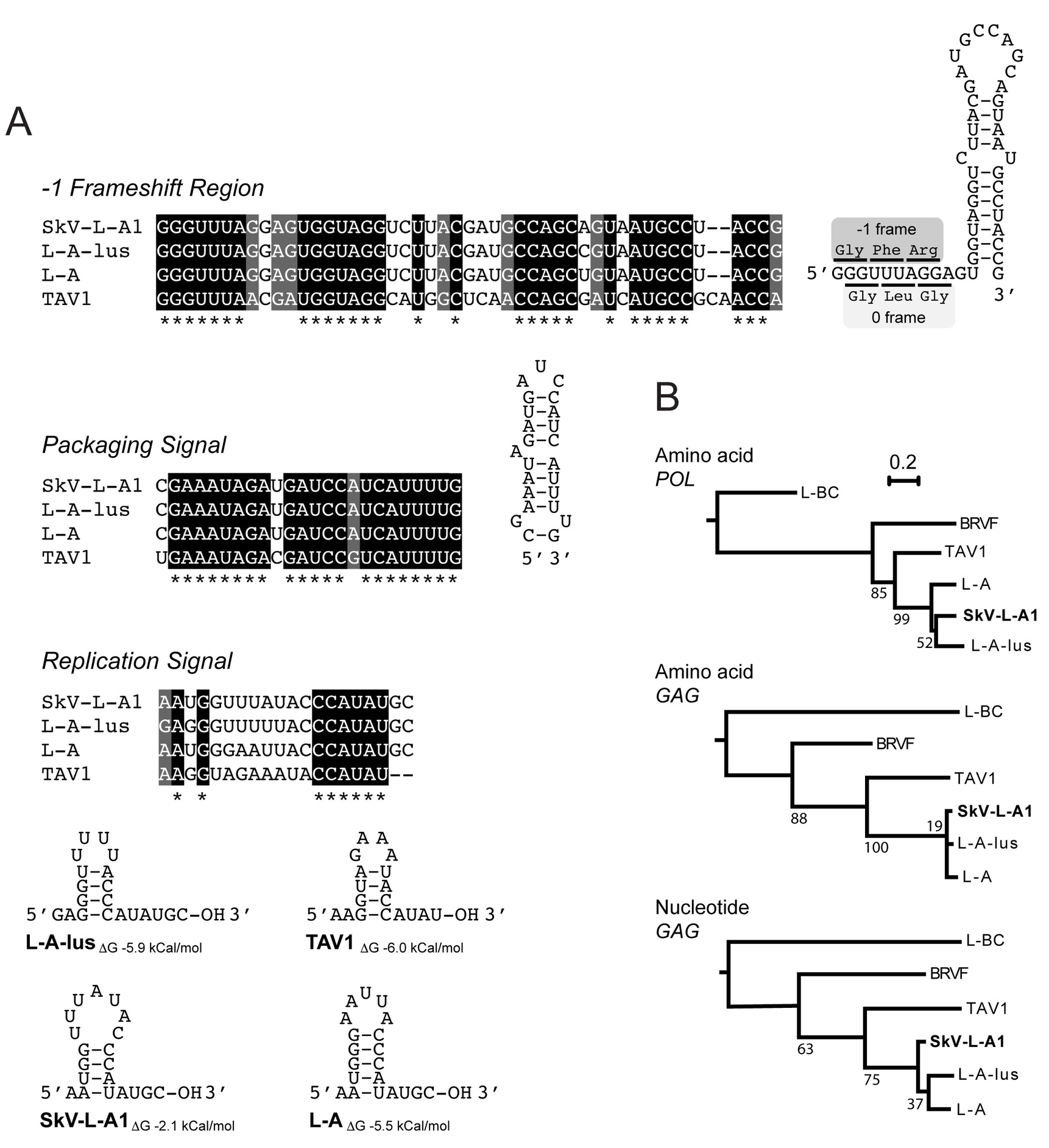

Supplement: S6 Fig — (A) RNA secondary structure models of functionally important totivirus RNA sequences are based upon the sequence of SkV-L-A1 unless otherwise stated, and show the predicted base pairing between nucleotides. (B) The evolutionary history of totivirus was inferred by using the Maximum Likelihood method with bootstrap values from 100 replicates shown at each node. The amino acid and nucleotide sequence of the POL and GAG gene from six totiviruses (GenBank accession numbers: SkV-L-A1 (this study; KX601068), L-A-lus (JN819511), L-A (NC_003745), tuber aestivum virus 1 (TAV1) (HQ158596), black raspberry virus F (BRVF) (NC_009890), L-BC (NC_001641)). The tree is drawn to scale, with branch lengths measured in the number of substitutions per site. (TIF) [file ppat.1005890.s006.tif]

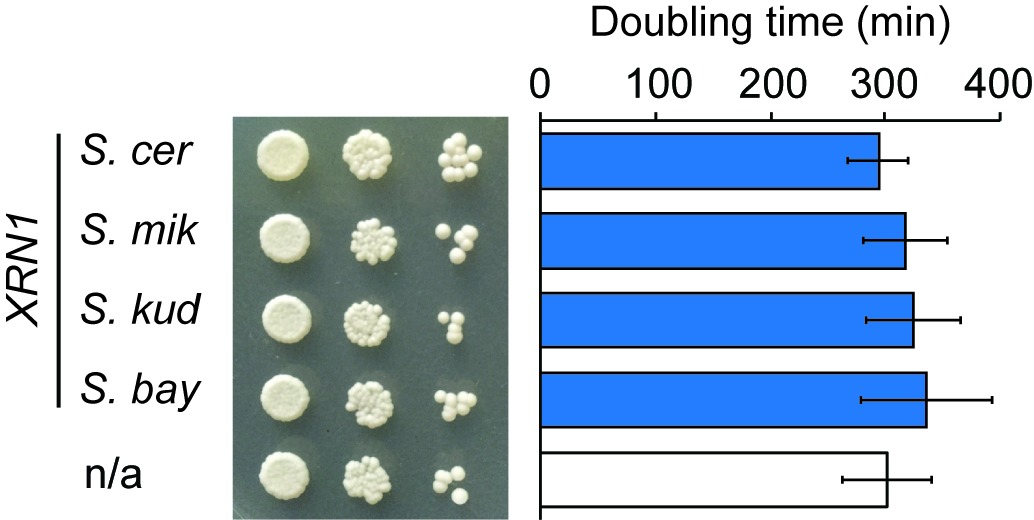

Supplement: S7 Fig — The growth of S. kudriavzevii expressing each of these XRN1 genes was measured by growing upon agar plates (left) or in liquid culture (right) and comparing to a wildtype strain that was not complemented with XRN1. (TIF) [file ppat.1005890.s007.tif]
