# Supplementary material for: XRN1 Is a Species-Specific Virus Restriction Factor in Yeasts
Source: PLoS Pathog. 2016 Oct 6;12(10):e1005890. doi: 10.1371/journal.ppat.1005890 (PMC5053509; doi:10.1371/journal.ppat.1005890)
Supplement: S1 Table — This table summarizes the results from all of the evolutionary analyses that were performed. (DOCX) [file ppat.1005890.s008.docx]

**Table S1. Evolutionary analysis of genes involved in RNA metabolism^a^**

|  | **PAML M7 vs M8^b^** | | | | | | **PAML M8 ^c^** | **REL ^c^** | **FEL^c^** | **MEME^c^** |  |
| --- | --- | --- | --- | --- | --- | --- | --- | --- | --- | --- | --- |
|  | Seq # ^d^ | ω0^e^ | codon freq.^f^ | 2*ΔInL^g^ | p-value^h^ | tree length^i^ | BEB pp>0.8 | BF> 50 | p<  0.05 | p< 0.05 |  |
| *CSL4* | 9  9  9  9 | 0.4  0.4  1.2  1.2 | f61  f3x4  f61  f3x4 | 0.00  0.00  0.00  0.00 | 1.00  1.00  1.00  1.00 | 2.39  2.36  2.39  2.36 | ns  ns  ns  ns | ns | ns | 214 |  |
| *SKI2* | 9  9  9  9 | 0.4  0.4  1.2  1.2 | f61  f3x4  f61  f3x4 | 0.01  0.00  0.02  0.02 | 1.00  1.00  0.99  0.99 | 2.44  2.35  2.44  2.35 | ns  ns  ns  ns | 50 | 50, 96 | 93, 297, 910 |  |
| *SKI3* | 8  8  8  8 | 0.4  0.4  1.2  1.2 | f61  f3x4  f61  f3x4 | 1.22  0.58  1.22  0.58 | 0.54  0.75  0.54  0.75 | 2.68  2.57  2.68  2.57 | 350, 351, 356, 585, 347, 350, 351, 585, 350, 351, 356, 585  347, 350, 351, 585 | ns | 351 | 351, 571, 697, 1375 |  |
| *SKI6* | 9  9  9  9 | 0.4  0.4  1.2  1.2 | f61  f3x4  f61  f3x4 | 0.00  0.00  0.00  0.00 | 1.00  1.00  1.00  1.00 | 1.90  1.85  1.90  1.85 | ns  ns  ns  ns | ns | ns | 134 |  |
| *SKI7* | 9  9  9  9 | 0.4  0.4  1.2  1.2 | f61  f3x4  f61  f3x4 | 4.73  3.88  4.73  0.01 | 0.09  0.14  0.09  0.99 | 3.70  3.52  3.70  3.50 | ns  ns  ns  ns | ns | 37, 71 | 71 |  |
| *SKI8* | 9  9  9  9 | 0.4  0.4  1.2  1.2 | f61  f3x4  f61  f3x4 | 2.34  2.75  2.34  2.75 | 0.31  0.25  0.31  0.25 | 2.21  2.16  2.21  2.16 | 108  108  108  108 | 102, 108, 117 | ns | ns |  |
| *XRN1* | 9 | 0.4 | f61 | 9.95 | **0.007** | 2.27 | 905, 1271, 1308, 1309, 1317, 1333, 1491, 1495, 1499 | 713, 827,  1338, 1503 | 827,  13331338 | 827, 1304, 1308, 1331, 1333, 1334, 1375 |  |
|  | 9 | 0.4 | f3x4 | 8.74 | **0.013** | 2.17 | 905, 1271, 1308, 1309, 1317, 1333, 1491, 1495, 1499 |  |  |  |  |
|  | 9 | 1.2 | f61 | 9.95 | **0.007** | 2.27 | 905, 1271, 1308, 1309, 1317, 1333, 1491, 1495, 1499 |  |  |  |  |
|  | 9 | 1.2 | f3x4 | 8.74 | **0.013** | 2.17 | 905, 1271, 1308, 1309, 1317, 1333, 1491, 1495, 1499 |  |  |  |  |

**Table S1 (continued)**

|  | **PAML M7 vs M8^b^** | | | | | | **PAML M8 ^c^** | **REL ^c^** | **FEL^c^** | **MEME^c^** |
| --- | --- | --- | --- | --- | --- | --- | --- | --- | --- | --- |
|  | Seq # ^d^ | ω0^e^ | codon freq.^f^ | 2*ΔInL^g^ | p-value^h^ | tree length^i^ | BEB pp>0.8 | BF> 50 | p< 0.05 | p< 0.05 |
| *DIS3* | 9  9  9  9 | 0.4  0.4  1.2  1.2 | f61  f3x4  f61  f3x4 | 2.21  2.62  2.21  2,62 | 0.33  0.27  0.33  0.27 | 2.25  2.14  2.25  2.14 | 580  580  580  580 | ns | ns | 240, 892 |
| *MTR3* | 9  9  9  9 | 0.4  0.4  1.2  1.2 | f61  f3x4  f61  f3x4 | 0.00  0.00  0.00  0.00 | 1.00  1.00  1.00  1.00 | 2.83  2.81  2.84  2.81 | ns  ns  ns  ns | ns | ns | ns |
| *RRP4* | 9  9  9  9 | 0.4  0.4  1.2  1.2 | f61  f3x4  f61  f3x4 | 0.01  0.00  0.01  0.00 | 1.00  1.00  1.00  1.00 | 2.43  2.36  2.43  2.36 | ns  ns  ns  ns | ns | ns | 50 |
| *RRP40* | 9  9  9  9 | 0.4  0.4  1.2  1.2 | f61  f3x4  f61  f3x4 | 7.04  7.32  7.04  7.32 | **0.03**  **0.03**  **0.03**  **0.03** | 2.55  2.49  2.55  2.49 | 10  10  10  10 | 10 | 10 | 10, 17, 201, 202 |
| *RRP42* | 9  9  9  9 | 0.4  0.4  1.2  1.2 | f61  f3x4  f61  f3x4 | 0.00  0.00  0.00  0.00 | 1.00  1.00  1.00  1.00 | 2.01  1.97  2.01  1.97 | ns  ns  ns  ns | ns | ns | ns |
| *RRP43* | 9  9  9  9 | 0.4  0.4  1.2  1.2 | f61  f3x4  f61  f3x4 | 0.03  0.03  0.03  0.03 | 1.00  1.00  1.00  1.00 | 2.36  2.31  2.36  2.31 | 328  328  328  328 | ns | ns | 63, 96, 311, 354 |
| *RRP45* | 9  9  9  9 | 0.4  0.4  1.2  1.2 | f61  f3x4  f61  f3x4 | 0.01  0.01  0.01  0.01 | 1.00  1.00  1.00  1.00 | 2.05  2.06  2.05  2.06 | ns  ns  ns  ns | ns | ns | ns |
| *RRP46* | 9  9  9  9 | 0.4  0.4  1.2  1.2 | f61  f3x4  f61  f3x4 | 0.00  0.00  0.00  0,00 | 1.00  1.00  1.00  1.00 | 2.39  2.31  2.39  2.31 | ns  ns  ns  ns | ns | ns | 77, 220 |

**Footnotes for Table S1**

**^a^** See methods section for a detailed account of experimental procedures and the following references for a more detailed explanation of the data presented in this table [47,94].

**^b^** In these columns, we compare two models of sequence evolution, one (M7) where positive selection is not allowed (dN/dS < 1) and one (M8) where positive selection is allowed, meaning that some codons that are allowed to have dN/dS > 1. The test is performed in the PAML program package.

**^c^** Codon positions in each gene identified with dN/dS > 1 by four different tests. Codons are reported in PAML M8 if they have a posterior probability >0.80 as calculated by the Bayes empirical Bayes methodology. The statistical cutoffs for reporting codons identified by REL, FEL, and MEME models (HyPhy package) are also given. Amino acids are numbered relative to *S. cerevisiae*.

**^d^** Number of *Saccharomyces* sequences used in the evolutionary analysis of each gene, consisting of sequences from *S. cerevisiae*, *S. paradoxus* (European, Far East, Hawaii and N. American isolates), *S. mikatae*, *S. kudriavzevii*, *S. arboricolus,* and *S. bayanus*.

**^e^** Initial seed value for ω (dN/dS) used in the maximum likelihood simulation.

**^f^** Model of codon frequency.

**^g^** Twice the difference in the natural logs of the likelihoods between M7 and M8 models. This value is used in a likelihood ratio test with two degrees of freedom.

**^h^** The p-value indicates the confidence with which the null model (M7) can be rejected.

**^i^** The tree length is the number of nucleotide substitutions per site along all branches in the phylogeny, and is a representation of total diversity in the dataset.
