# Supplementary material for: XRN1 Is a Species-Specific Virus Restriction Factor in Yeasts
Source: PLoS Pathog. 2016 Oct 6;12(10):e1005890. doi: 10.1371/journal.ppat.1005890 (PMC5053509; doi:10.1371/journal.ppat.1005890)
Supplement: S2 Table — This table lists information on the various plasmids constructed and/or used in this study. (DOCX) [file ppat.1005890.s009.docx]

**Table S2 – Relevant plasmids.**

| ***Plasmid*** | ***Details*** | ***Markers*** | ***Source*** |
| --- | --- | --- | --- |
| pPAR219 | *XRN1 S. cerevisiae* | *LEU2*, *2μm* | This study |
| pPAR225 | *XRN1 S. mikatae* | *LEU2*, *2μm* | This study |
| pPAR226 | *XRN1 S. bayanus* | *LEU2*, *2μm* | This study |
| pPAR227 | *XRN1 S. kudriavzevii* | *LEU2*, *2μm* | This study |
| pPAR221 | *NUP133 S. cerevisiae* | *LEU2*, *2μm* | This study |
| pPAR267 | Chimeric *XRN1* (Sc-775) | *LEU2*, *2μm* | This study |
| pPAR266 | Chimeric *XRN1* (Sc-461) | *LEU2*, *2μm* | This study |
| pPAR274 | Chimeric *XRN1* (Sc-241) | *LEU2*, *2μm* | This study |
| pPAR277 | Chimeric *XRN1* (Sc-D1-D3) | *LEU2, 2μm* | This study |
| pPAR278 | Chimeric *XRN1* (Sc-D1) | *LEU2, 2μm* | This study |
| pPAR289 | Chimeric *XRN1* (Sc-D2+D3) | *LEU2, 2μm* | This study |
| pPAR288 | *XRN1 S. kudriavzevii* (T827F) | *LEU2, 2μm* | This study |
| pPAR290 | Chimeric *XRN1* (Sk-777) | *LEU2, 2μm* | This study |
| pPAR100 | *GAL*-eGFP | *URA3, 2μm* | This study |
| pPAR281 | *GAL-XRN1* *S. cerevisiae* | *URA3, 2μm* | This study |
| pPAR282 | *GAL-XRN1* *S. mikatae* | *URA3, 2μm* | This study |
| pPAR283 | *GAL-XRN1 S. kudriavzevii* | *URA3, 2μm* | This study |
| pPAR284 | *GAL-XRN1 S. bayanus* | *URA3, 2μm* | This study |
| pPAR264 | *XRN1(HA) S. cerevisiae* | *LEU2, 2μm* | This study |
| pPAR285 | *XRN1(HA) S. mikatae* | *LEU2, 2μm* | This study |
| pPAR286 | *XRN1(HA) S. bayanus* | *LEU2, 2μm* | This study |
| pPAR287 | *XRN1(HA) S. kudriavzevii* | *LEU2, 2μm* | This study |
| pPAR301 | *XRN1(HA) S. cerevisiae* | *TRP1*, *2μm* | This study |
| pPAR302 | *XRN1(HA) S. mikatae* | *TRP1*, *2μm* | This study |
| pPAR303 | *XRN1(HA) S. kudriavzevii* | *TRP1*, *2μm* | This study |
| pPAR304 | *XRN1(HA) S. bayanus* | *TRP1*, *2μm* | This study |
| pPAR181 | *MET17-*FLAG | *TRP1*, *2μm* | This study |
| pPAR330 | *GAL*-Gag(L-A)-V5 | *TRP1, CEN* | This study |
| pPAR331 | *GAL*-Gag(L-A)-myc | *TRP1, CEN* | This study |
| pGTy1HIS3(AI) | *GAL-1* inducible Ty1(*HIS3*(AI)) | *URA3, 2μm* | Curcio *et al.* [89] |
| pI2L2 | L-A cDNA expression plasmid | *TRP1* | Ribas et al. [88] |
| pRDK307 | *XRN1 S. cerevisiae* | *LEU2, CEN* | Page et al. [54] |
| pRDK307-Δ1493-1528 | *XRN1 S. cerevisiae* C- terminus truncation | *LEU2, CEN* | Page et al. [54] |
| pRDK307-Δ1392-1528 | *XRN1 S. cerevisiae* C-terminus truncation | *LEU2, CEN* | Page et al. [54] |
| pRDK307-Δ1206-1528 | *XRN1 S. cerevisiae* C- terminus truncation | *LEU2, CEN* | Page et al. [54] |
| pAJ152 | *XRN1 S. cerevisiae* | *LEU2, CEN* | Page et al. [54] |
| pAJ152-E176G | *XRN1*(E176G) *S. cerevisiae* | *LEU2, CEN* | Page et al. [54] |
