# Supplementary material for: XRN1 Is a Species-Specific Virus Restriction Factor in Yeasts
Source: PLoS Pathog. 2016 Oct 6;12(10):e1005890. doi: 10.1371/journal.ppat.1005890 (PMC5053509; doi:10.1371/journal.ppat.1005890)
Supplement: S3 Table — This table lists information on the various Saccharomyces strains constructed and/or used in this study. (DOCX) [file ppat.1005890.s010.docx]

**Table S3 – Relevant yeast strains and species.**

| ***Strain*** | ***Genotype*** | ***Source*** |
| --- | --- | --- |
| S288C | MATα *SUC2 gal2 mal2 mel flo1 flo8-1 hap1 ho bio1 bio6* [L-A] |  |
| BY4741 | MATa *his3*Δ*1 leu2*Δ*0 met15*Δ*0 ura3*Δ*0*  [L-A] |  |
| BY4741 *ski2*Δ | MATa *his3*Δ*1 leu2*Δ*0 met15*Δ*0 ura3*Δ*0 ski2*Δ [L-A] |  |
| BY4741 *ski3*Δ | MATa *his3*Δ*1 leu2*Δ*0 met15*Δ*0 ura3*Δ*0 ski3*Δ [L-A] |  |
| BY4741 *ski8*Δ | MATa *his3*Δ*1 leu2*Δ*0 met15*Δ*0 ura3*Δ*0 ski8*Δ [L-A] |  |
| BY4741 *xrn1*Δ | MATa *his3*Δ*1 leu2*Δ*0 met15*Δ*0 ura3*Δ*0 xrn1*Δ [L-A] |  |
| BY4733 | MATa *his3*Δ*200 trp1*Δ*63 leu2*Δ*0 met15*Δ*0 ura3*Δ*0* [L-A] |  |
| AKU-4011 (K12) | Diploid sake yeast | J. Fay |
| 1368 | MATα *his4 kar1* [L-A, M] | R. Wickner |
| 2404 | MATα *his4 kar1* [L-A] | R. Wickner |
| 2405 | MATα *his4 kar1* | R. Wickner |
| BJH001 | MATa *his3*Δ*200 leu2*Δ*0 met15*Δ*0 trp1*Δ*63 ura3*Δ*0* [L-A, M] | This study |
| BJH006 | MATa *his3*Δ*200 leu2*Δ*0 met15*Δ*0 trp1*Δ*63 ura3Δ0 xrn1*Δ::*KANMX4* [L-A, M] | This study |
| *S. mikatae* NBRC1815 | Type strain | E. Louis |
| *S. bayanus* CBS7001 | Type strain | E. Louis |
| *S. kudriavzevii* NBRC1802 | Type strain | E. Louis |
| *S. mikatae* JRY9181 | MATa *ho*Δ::*KANMX trp1*Δ::*HYGMX* *ura3*Δ::*HYGMX* | C.T. Hittinger [46] |
| *S. bayanus* JRY8153 | MATa *ho*Δ::*NAT*, *his3-1, lys2-5, trp-1, ura3-1* | C.T. Hittinger [46] |
| *S. kudriavzevii* FM1183 | MATa *ho*Δ::*KANMX ura3Δ0 trp1Δ0* [SkV-L-A1] | C.T. Hittinger [46] |
